# Shared sex hormone metabolism-related gene prognostic index between breast and endometrial cancers

**DOI:** 10.3389/fendo.2023.1126862

**Published:** 2023-01-20

**Authors:** Junyi Duan, Chenan Liu, Jiahong Yi, Yun Wang

**Affiliations:** ^1^ First Clinical Medical College, Shanxi Medical University, Taiyuan, China; ^2^ Department of Gastrointestinal Surgery, Beijing Shijitan Hospital, Capital Medical University, Beijing, China; ^3^ Department of Clinical Nutrition, Beijing Shijitan Hospital, Capital Medical University, Beijing, China; ^4^ Sun Yat-Sen University Cancer Center, Sun Yat-Sen University, Guangzhou, China; ^5^ Department of Obstetrics and Gynecology, The 985th Hospital of The People’s Liberation Army Joint Logistic Support Force, Taiyuan, China

**Keywords:** endometrioid endometrial cancer, breast cancer, sex hormone metabolism-related gene, prognostic index, weighted gene co-expression network analysis, immunotherapy

## Abstract

**Aims:**

As sex hormone-dependent tumors, it remains to be clarified whether there is a common genetic signature and its value between breast and endometrial cancers. The aim of this study was to establish the shared sex hormone metabolism-related gene prognostic index (SHMRGPI) between breast and endometrial cancers and to analyze its potential role in the therapeutic and prognostic assessment of endometrial cancers.

**Methods:**

Using transcriptome data from TCGA, tumor-associated gene modules were identified by weighted gene co-expression network analysis, and the intersection of module genes with female sex hormone synthesis and metabolism genes was defined as sex hormone metabolism-related gene. SHMRGPI was established by the least absolute shrinkage and selection operator and Cox regression. Its prognostic value of patients with endometrial cancer was validated, and a nomogram was constructed. We further investigated the relationship between SHMRGPI groups and clinicopathological features, immune infiltration, tumor mutation burden, and drug sensitivity.

**Results:**

A total of 8 sex hormone metabolism-related gene were identified as key genes for the construction of prognostic models. Based on SHMRGPI, endometrial cancer patients were divided into high and low SHMRGPI groups. Patients in the low SHMRGPI group had longer overall survival (OS) compared with the high group (*P*< 0.05). Furthermore, we revealed significant differences between SHMRGPI groups as regards tumor immune cell infiltration, somatic mutation, microsatellite instability and drug sensitivity. Patients with low SHMRGPI may be the beneficiaries of immunotherapy and targeted therapy.

**Conclusions:**

The SHMRGPI established in this study has prognostic power and may be used to screen patients with endometrial cancer who may benefit from immunotherapy or targeted therapy.

## 1 Introduction

Breast and endometrial cancers are the first and fourth most common tumors in women and pose a serious threat to women’s health (1). As tumors originating from sex hormone-dependent organs, there is accumulating evidence that sex hormones and dysregulated hormonal signaling influence disease origin, treatment response, and clinical outcomes in breast and endometrial cancers (2–6). Based on clinicopathological characteristics and immunohistochemical tests, breast cancer has been classified into different pathological subtypes, in which patients who are estrogen receptor (ER) and progesterone receptor (PR) positive show an ideal response to endocrine therapy and a good prognosis (7). Similarly, endometrioid endometrial cancer (EEC), as a tumor affected by sex hormones and sensitive to endocrine therapy (8, 9). Investigating the molecular markers and potential mechanisms it shares with breast cancer can help deepen our understanding of EEC.

The morbidity and mortality of endometrial cancer have increased annually in recent years, which is probably due to the combined effects of an aging population, the decline in benign hysterectomies, and the obesity epidemic (10). Despite significant advances in various aspects of endometrial cancer management, the cumulative threat to women’s health from endometrial cancer has not abated. Molecular classification based on genomic features has improved our understanding of endometrial cancer and clinical practice has changed as a result (11). Further analysis of The Cancer Genome Atlas (TCGA) data to advance our knowledge of the tumor and address the rising burden of disease is critical.

In this study, we performed a weighted gene co-expression network analysis (WGCNA) using transcriptome data from the TCGA database for breast and endometrial cancers to identify the sex hormone metabolism-related gene (SHMRG) associated with the synthesis and metabolism of female sex hormone, and subsequently established the SHMRG prognostic index (SHMRGPI) in patients with endometrial cancer and analyzed the value of SHMRGPI in survival assessment and therapeutic modality selection. With this study, we hope to identify novel biomarkers that can be used for screening and treatment and provide a basis for the search for potential beneficiaries of immunotherapy and targeted therapy.

## 2 Methods

### 2.1 Data download and selection

The transcriptome data for the breast cancer samples were downloaded from TCGA. Samples from ER and PR positive female patients were selected for subsequent analysis. Transcriptome, somatic variation and clinical data for endometrial cancer samples were downloaded from TCGA, and EEC samples were selected. Transcriptome data for normal breast and uterine tissues were downloaded from the Genotype-Tissue Expression (GTEx) dataset from XENA (12). Gene sets associated with the synthesis and metabolism of female sex hormones were downloaded from the Molecular Signatures Database (MSigDB) for subsequent analysis (13).

### 2.2 Identification of SHMRG

Weighted gene co-expression network analysis (WGCNA) was established to explore the relationship between expression and phenotype data based on correlation coefficients (14). In this study, WGCNA was used to identify gene modules associated with ER/PR positive breast cancer and EEC. First, the sample clustering was performed and abnormal samples were removed. After correlations between genes were calculated, a matrix was built for gene stratification and module clustering to determine the correlation between gene modules and tumors based on the eigenvalues of gene modules of tumor samples and normal samples. In the WGCNA of this study, the soft threshold beta was 2, minModuleSize was 30, mergeCutHeight was 0.25 and deepSplit was 2. Subsequently, we took the intersection of EEC-related modules, ER/PR positive breast cancer-related modules and gene sets downloaded from MSigDB as shared SHMRG.

### 2.3 Training and testing of the SHMRGPI

The EEC sample was randomly divided into training and testing cohorts in a ratio of 7:3. SHMRG associated with the prognosis of EEC patients was screened in the training cohort using univariate Cox regression. Subsequently, the SHMRGPI was established using the least absolute shrinkage and selection operator (LASSO) and stepwise multivariate Cox regression. The SHMRGPI was calculated according to the following equation:


$$\text{SHMRGPI}=\sum_{i}\text{coefficient}({\text{SHMRG}}_{i})\times \text{expression}({\text{SHMRG}}_{i})$$

The distributions of SHMRGPI and survival status were plotted as scatter plots. The correlation between SHMRG and SHMRGPI was shown as heat maps. Patients with EEC were divided into two groups based on the median SHMRGPI (**Supplementary Table 1**). The principal component analysis (PCA) showed the distribution of the two SHMRGPI groups. Overall survival (OS) was compared between the two groups using the log-rank test. We plotted time-dependent receiver operating characteristic (tdROC) curves and calculated the area under the curve (AUC) for assessing the predictive power of SHMRGPI. The above analysis was validated in the test and entire cohorts. The SHMRGPI was established and validated using survminer, survival, glmnet, ggplot2 R packages.

### 2.4 Clinical correlation of the prognostic model

We performed a multifactor ROC analysis to demonstrate the advantages of SHMRGPI over other relatively complete clinicopathological characteristics (age, tumor stage and grade) in prognostic prediction. We then use the regplot R packages to develop a nomogram. The accuracy of the nomogram was assessed using calibration curves. Furthermore, we performed a stratified analysis to examine the predictive power of SHMRGPI in different clinical subgroups.

### 2.5 Immune correlation analysis

To assess immune cell infiltration in the different SHMRGPI groups, we performed single sample gene set enrichment analysis (ssGSEA). Then we investigated the differential expression of immune checkpoint genes between the SHMRGPI groups. Tumor immune dysfunction and exclusion (TIDE) scores predict the response of immunotherapy by model primary mechanisms of tumor immune evasion (15). We calculated TIDE scores for each sample using an online tool to analyze the differences in TIDE scores between SHMRGPI groups.

### 2.6 Tumor somatic mutation analysis

We used the maftools R package to collate and analyze the somatic mutation data from patients with EEC. Fifteen genes with the highest tumor mutation frequency in each SHMRGPI group were visually analyzed. The tumor mutation burden (TMB) was subsequently calculated for each sample to analyze the discrepancy in TMB levels between SHMRGPI groups. After establishing subgroups based on the median TMB, we analyzed the prognostic value of SHMRGPI in the TMB subgroups. The microsatellite instability (MSI) status of EEC patients is downloaded by invoking the cBioPortalData R package. Thereafter, differences of MSI status in the SHMRGPI group and differences of SHMRGPI in the MSI subgroup were analyzed.

### 2.7 Drug sensitive analysis

The half-maximal inhibitory concentrations (IC50) of common antitumor drugs were predicted for both SHMRGPI groups based on data from Cancer Drug Sensitivity Genomics (16). The differences in IC50 between the two groups were analyzed and visualized by the oncopredict and ggplot2 R packages (17).

### 2.8 Statistical analysis

The WGCNA was analyzed and visualized using the WGCNA and limma R packages. Cox regression and survival analysis was performed by the survivor and survminer R packages. Differences in survival between groups were visualized using Kaplan-Meier survival curves. The Wilcoxon signed-rank test was used to test the differences between quantitative data. The entire analysis was conducted in R (version 4.0.3). *P*< 0.05 was considered statistically significant.

## 3 Results

### 3.1 The tumor-related gene modules in ER/PR positive breast cancer and EEC

**Figure 1:**
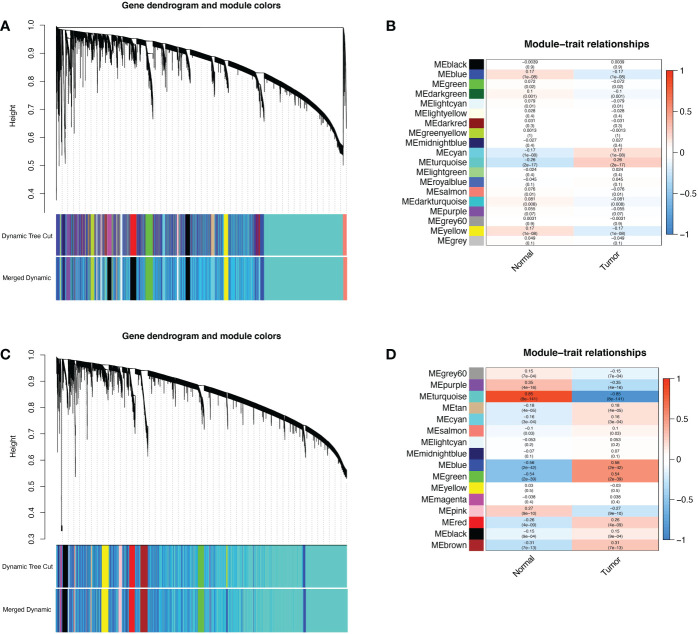
WGCNA of ER/PR-positive breast cancer and EEC. **(A)** The cluster dendrogram of ER/PR-positive breast cancer. **(B)** Correlation of WGCNA modules and ER/PR-positive breast cancer. **(C)** The cluster dendrogram of ER/PR-positive breast cancer. **(D)** Correlation of WGCNA modules and EEC. WGCNA, weighted gene co-expression network analysis. ER, estrogen receptor; PR, progesterone receptor.

802 ER/PR positive tumor tissue samples and 79 normal samples were selected from the TCGA-BRCA dataset, and 179 normal samples from the GTEx database were combined for WGCNA after batch effects were removed. The gene clustering dendrogram was shown in **Figure 1A**. The expression matrix was divided into 19 gene modules, and four modules “blue”, “cyan”, “turquoise” and “yellow” were highly associated with ER/PR positive breast cancer and identified as ER/PR positive breast cancer-related modules (**Figure 1B**). Similarly, 408 EEC tissue samples and 19 normal samples were selected from the TCGA-UCEC dataset, and 78 normal samples from the GTEx database were combined. The results of the WGCNA after removing batch effects were showen as in **Figures 1C, D**. The expression matrix was divided into 16 gene modules, “grey”, “purple”, “turquoise”, “tan”, “cyan”, “blue”, “green”, “pink”, “red”, “black”, “brown” were highly associated with EEC and were identified as EEC-related modules.

### 3.2 Identification of shared SHMRG

The female sex hormone synthesis and metabolism related-gene sets downloaded from MSigDB were shown in **Supplementary Table 2**. The intersection of ER/PR positive breast cancer-related modules, EEC-related modules, and gene sets from MSigDB was taken. As a result, 126 genes were identified as shared SHMRG (**Supplementary Table 3**).

### 3.3 Training and testing of SHMRGPI

**Figure 2:**
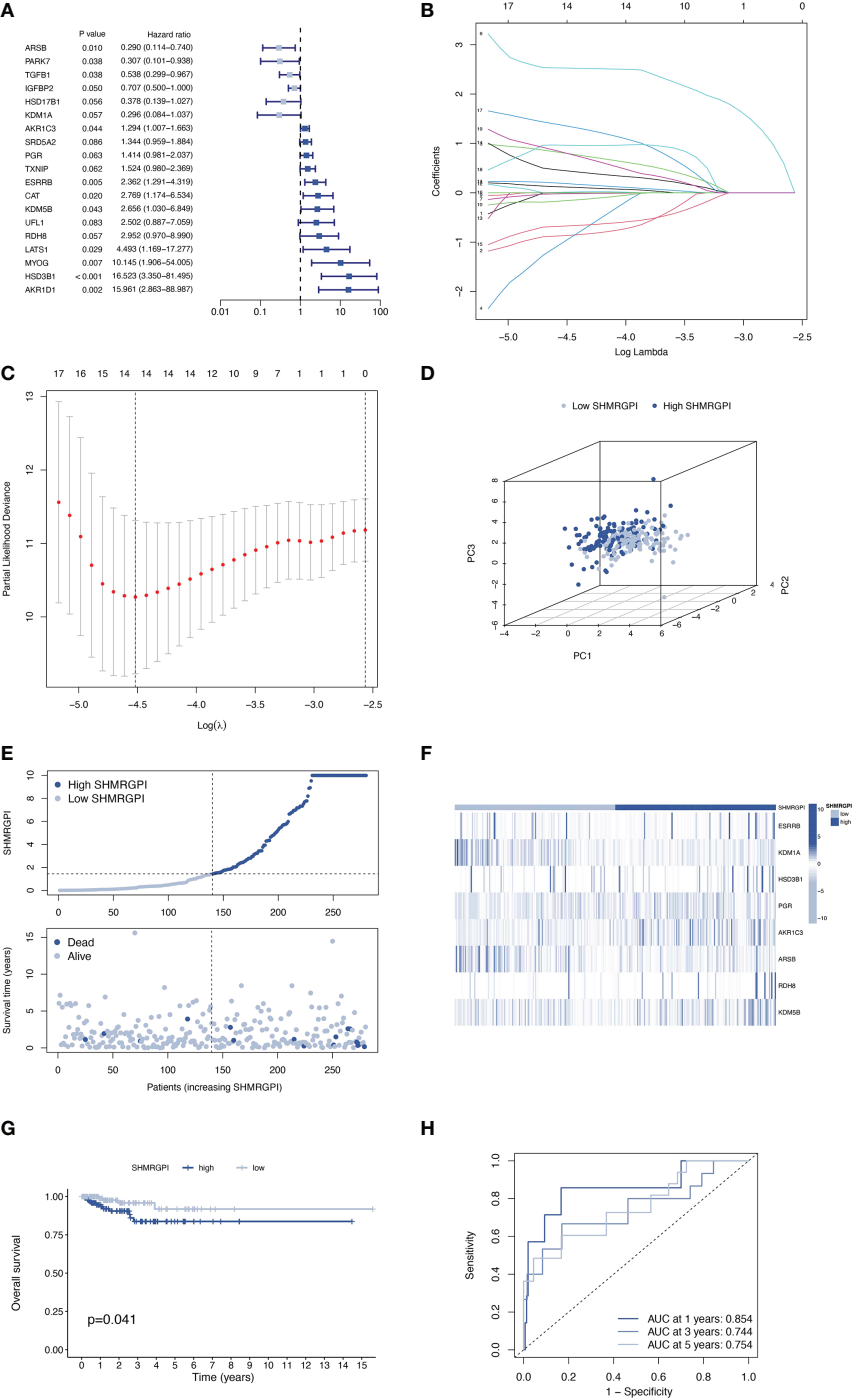
Establishment of the SHMRGPI in the train cohort. **(A)** Univariate Cox regression analysis for screening prognostic SHMRG. **(B, C)** LASSO regression analysis for variable selection. **(D)** PCA plot for different SHMRGPI groups. **(E)** Scatter diagram for the SHMRGPI and survival status of EEC patients. **(F)** Heat map for the expression of SHMRG and SHMRGPI groups. **(G)** Kaplan–Meier curves of survival difference between SHMRGPI groups. **(H)** ROC for predicting the sensitivity and specificity of survival according to the SHMRGPI. SHMRGPI, sex hormone metabolism-related gene prognostic index; LASSO, least absolute shrinkage and selection operator; PCA, principal component analysis; EEC, endometrioid endometrial cancer. ROC, receiver operating characteristic curve.

Total 399 samples from the TCGA database were included in this study and were randomly divided into training and test groups in a 7:3 ratio. The univariate regression analysis of SHMRG combined with expression and clinical data revealed a total of 19 SHMRG were potentially associated with prognosis in patients with EEC (*P<* 0.1, **Figure 2A**). We then performed LASSO and stepwise multivariate Cox regression, and finally identified 8 SHMRG for the construction of the SHMRGPI (**Figures 2B, C**). The SHMRGPI was calculated according to the following formula, and the training cohort was divided into high and low groups based on the median SHMRGPI:

SHMRGPI = *ESRRB* × 1.42469 – *KDM*1*A* × 3.68341 + *HSD*3*B*1 × 3.21536 + *PGR* × 0.52695 + *AKR*1*C*3 × 0.38220 – *ARSB* × 1.10113 + *RDH*8 × 2.77368 + *KDM*5*B* × 2.34959.

PCA analysis showed the difference in distribution between the two SHMRGPI groups (**Figure 2D**). The survival time and survival status of the SHMRGPI groups in patients with EEC were shown in **Figure 2E**. As SHMRGPI increased, survival time decreased and the number of deaths increased. The expression of key genes of SHMRGPI and their correlation with SHMRGPI groups were shown in a heat map (**Figure 2F**). The Kaplan-Meier curves demonstrated a better prognosis in the low SHMRGPI group (P = 0.041, **Figure 2G**). The tdROC illustrated the predictive power of SHMRGPI (AUC 0.854 at one year, 0.744 at three years, and 0.754 at five years, **Figure 2H**).

**Figure 3:**
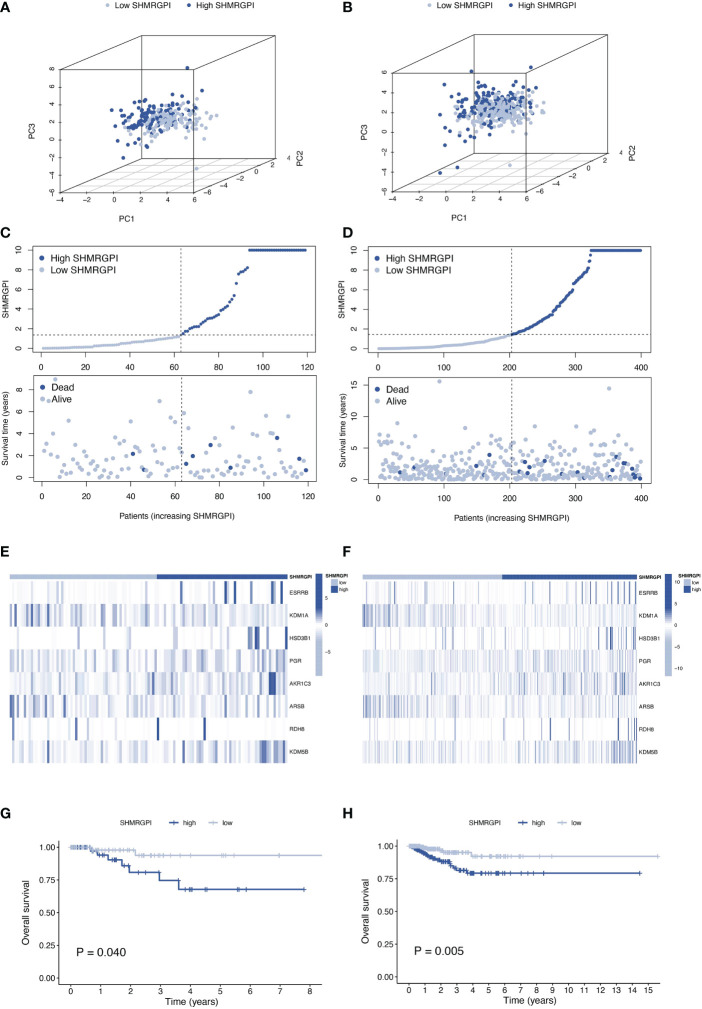
Validation of the SHMRGPI in the test and entire cohorts. PCA plot **(A)**, scatter plot **(B)**, heat map **(C)** and Kaplan–Meier curves **(D)** for the test cohort. PCA plot **(E)**, scatter plot **(F)**, heat map **(G)**, and Kaplan–Meier curves **(H)** for the entire cohort.

The same distribution between groups and differential survival results as in the training cohort were also observed in the test and entire cohorts, validating the strong and robust prognostic power of SHMRGPI (**Figures 3A-H**).

### 3.4 Correlation analysis of clinical features

**Figure 4:**
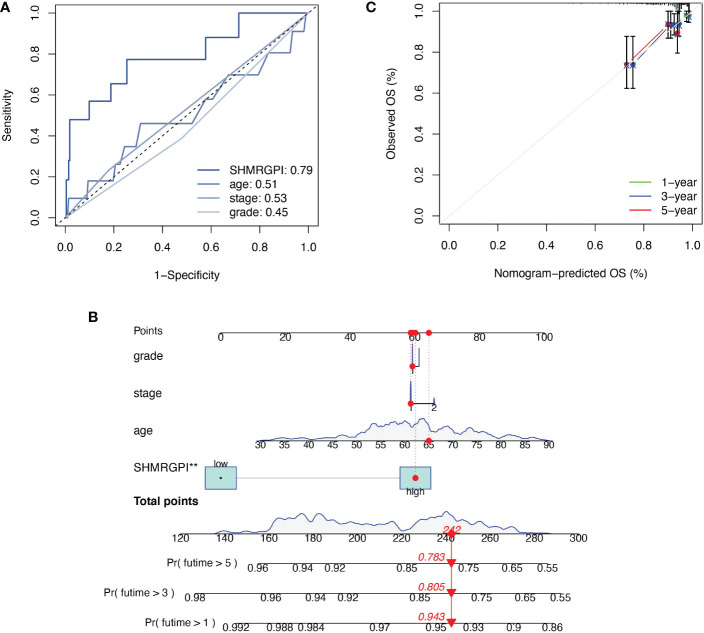
Construction and examination of nomogram. **(A)** The multifactor ROC of SHMRGPI, age, FIGO stage, and tumor grade. **(B)** The nomogram for predicting prognosis of EEC patients. **: p value < 0.01. **(C)** The calibration curves of the nomogram. ROC, receiver operating characteristic curve.

**Figure 5:**
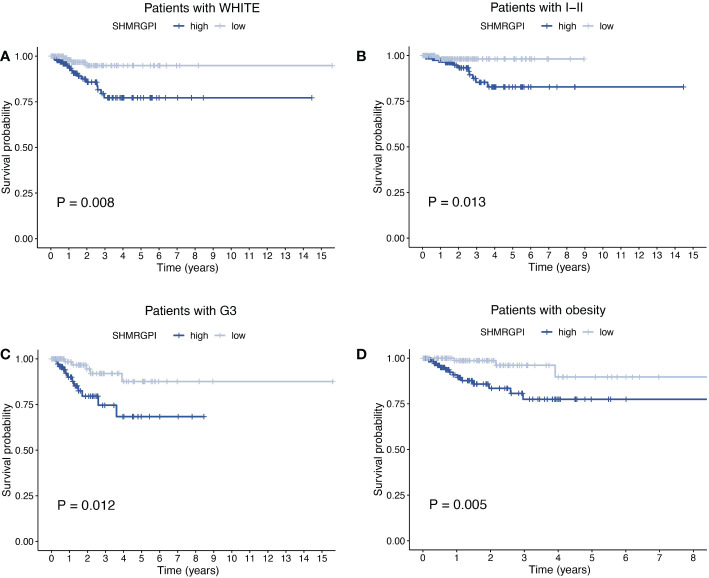
Stratified analysis of EEC patients. Kaplan-Meier curves of patients with white race **(A)**, FIGO stage I-II **(B)**, tumor grade 3 **(C)**, BMI ≥ 30 **(D)**. FIGO, the international federation of gynecology and obstetrics. BMI, body mass index.

The Multifactor ROC analysis demonstrated the strong predictive power of SHMRGPI compared to other clinicopathological (**Figure 4A**). Combining SHMRGPI, age, tumor stage, and grade, we constructed a nomogram to predict survival at one, three, and five years after diagnosis in EEC patients. (**Figure 4B**). The agreement between the predictions of the nomogram and actual observations was confirmed using calibration curves (**Figure 4C**). To further assess the prognostic value of SHMRGPI, we performed a stratified analysis in the entire cohort. The results showed that SHMRGPI was associated with prognosis in white, FIGO stage I-II, tumor grade 3 and obese EEC patients (*P*< 0.05; **Figures 5A-D**).

### 3.5 Immune correlation analysis

**Figure 6:**
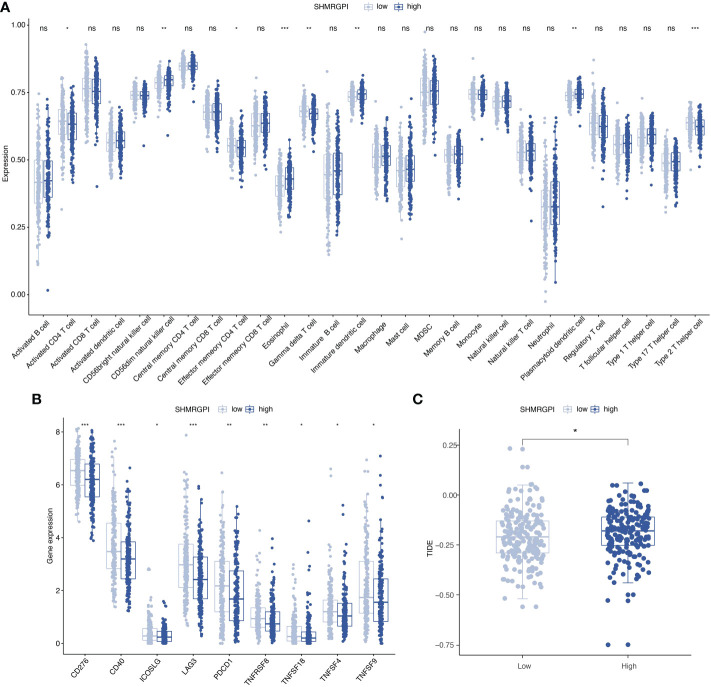
Immune correlation analysis. **(A)** Comparison of the discrepancy in immune cell infiltration between two groups based on ssGSEA. **(B)** Differences in the expression of immune checkpoint genes between the two groups. **(C)** Differences in the TIDE scores between the two groups. ssGSEA, single-sample gene set enrichment analysis; TIDE, tumor immune dysfunction and exclusion. ns, not significant. *: p value < 0.05, **: p value < 0.01, and ***: p value < 0.001.

To investigate the relationship between SHMRGPI grouping and immune status, ssGSEA analysis was performed for each immune cell subset. Activated CD4+ T cells, effector memory CD4+ T cells, gamma delta T cells, and type 2 T helper cells were more infiltrated in the low SHMRGPI group, whereas CD56dim natural killer cells, eosinophil Immature dendritic cells, and plasmacytoid dendritic cells were more infiltrated in the high SHMRGPI group (**Figure 6A**). To explore the potential value of SHMRGPI in immunotherapy, we further analyzed the expression of immune checkpoint genes between the two groups and found that the immune checkpoint genes *CD276*, *CD40*, *ICOSLG*, *LAG3*, *PD-1*, *TNFRSF8*, *TNFSF4*, *TNFSF9*, and *TNFSF18* were more highly expressed in the low SHMRGPI group (**Figure 6B**). In addition, we predicted the response of each SHMRGPI group to immunotherapy based on the TIDE scores, and the results showed that the low SHMRGPI group was more likely to benefit from immunotherapy (**Figure 6C**).

### 3.6 Gene mutation analysis

**Figure 7:**
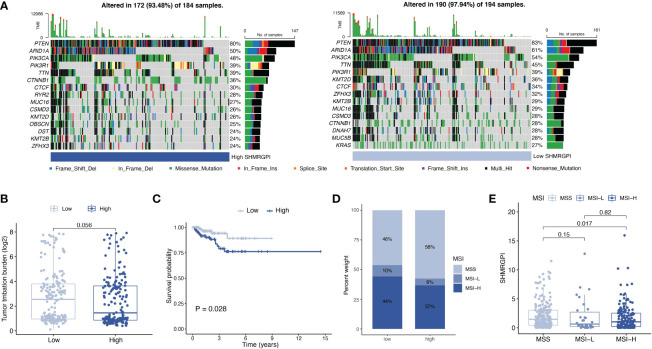
Analysis of mutation data. **(A)** Waterfall plot for somatic mutations in different SHMRGPI groups. **(B)** Differential analysis of TMB in different SHMRGPI groups. **(C)** Kaplan–Meier curves of survival differences between the SHMRGPI groups in patients with low TMB. **(D)** Distribution of MSI status in the different SHMRGPI groups. **(E)** Differential analysis of the SHMRGPI in patients with different MSI status. TMB, tumor mutation burden; MSI, microsatellite instability.

Somatic mutations in different SHMRGPI groups were demonstrated by waterfall plots (**Figure 7A**). *PTEN*, *ARID1A*, *PIK3CA*, and TTN were mutated frequently in the EEC and more frequently in the low SHMRGPI group, and *CTNNB1* was mutated more frequently in the high SHMRGPI group. After that, we calculated the tumor mutation burden in each group and observed difference close to the statistical threshold in TMB levels between SHMRG groups, with relatively higher TMB in the low SHMRGPI group (**Figure 7B**). Grouped by median TMB, SHMRGPI maintained its prognostic value in the low TMB subgroup (**Figure 7C**). We further analyzed the correlation between MSI and SHMRGPI. **Figure 7D** shows the difference in the distribution of microsatellite instability status in SHMRGPI groups, with a higher proportion of MSI-high (MSI-H) in patients in the low SHMRGPI group. Patients with MSI-H patients had a lower SHMRGPI, compared to patients with microsatellite stable (MSS, **Figure 7E**).

### 3.7 Drug sensitive analysis

**Figure 8:**
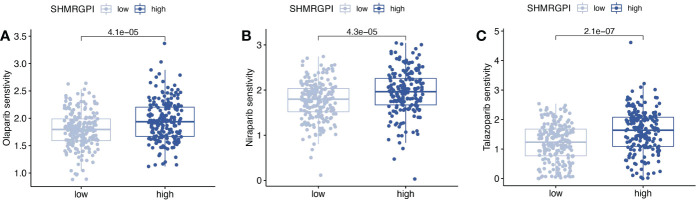
Drug sensitivity analysis. Comparison of drug sensitivity differences between SHMRGPI groups in olaparib **(A)**, niraparib **(B)**, and talazoparib **(C)**.

Drug sensitivity analysis based on the oncopredict R package revealed significant differences between the SHMRGPI groups in olaparib, niraparib, and talazoparib (**Figures 8A–C**). The low SHMRGPI group was more sensitive (*P*< 0.05).

## 4 Discussions

Sex hormones play an important role in the oncogenesis, diagnosis, and treatment of breast cancer, and similarly, sex hormones influence the management of patients with endometrial cancer as important disease-related risk factors (7, 9, 10). However, few studies have addressed the transcriptomic commonalities between breast and endometrial cancers. Our study used WGCNA to identify shared SHMRG between ER/PR-positive breast cancer and EEC. On this basis, we explore the potential benefits of SHMRG in the management of patients with EEC.

By Cox and LASSO regression we established a prognostic gene formula called SHMRGPI, which includes *ESRRB*, *KDM1A*, *HSD3B1*, *PGR*, *AKR1C3*, *ARSB*, *RDH8*, and *KDM5B*. *KDM1A*, also called *LSD1*, has been found to be aberrantly expressed in a variety of cancers and is closely associated with cellular effects such as epithelial-mesenchymal transition (EMT), proliferation, and malignant transformation (18). Drugs targeting *KDM1A* have entered clinical studies in small cell lung cancer and acute myelocytic leukemia (19). In the treatment of endometrial cancer, combined with mTOR inhibitors, *KDM1A* inhibitors were found to inhibit tumor growth in ex vivo and *in vivo* experiments (20). The protein encoded by *PGR*, a member of the steroid receptor superfamily, regulates the biological effects of progesterone, and it has been studied as an important marker associated with prognosis and disease progression in endometrial cancer (21). *KDM5B*, *ARSB*, *AKR1C3*, *HSD3B1*, *ESRRB* are involved in steroid hormone metabolism and have been found to be associated with other hormone-dependent tumors, such as prostate and breast cancers (22–26). However, evidence of their association with endometrial cancer is limited. The enzyme encoded by RDH8 involved in the rhodopsin regeneration pathway, but it’s relationship with tumors has not been mentioned.

Based on SHMRGPI, EEC patients were divided into two distinct groups. The prognostic value of SHMRGPI for patients with EEC was demonstrated from different points of view. Combining age, FIGO stage, tumor grade, and SHMRGPI, we developed a nomogram to stratify the prognosis of patients with EEC, which may have clinical significance.

Considering the complex relationship between host immune function and tumor, and the prospect of immunotherapy in the treatment of patients with EEC, we analyzed the discrepancy in immune cell infiltration in each SHMRGPI group. The results revealed that CD4+ T cells, Gamma delta T cells, and Type 2 T helper cells were more abundant in the low SHMRGPI group. CD4+ T cells and TH cells played an important supportive role in the anti-tumor immune effect, and Gamma delta T cells killed tumor cells through a non-MHC-restricted manner (27, 28). Their infiltration reflects the active anti-tumor immune effect in the low SHMRGPI group. Subsequently, we analyzed the expression of immune checkpoint genes in different SHMRGPI groups and found that a variety of immune checkpoint genes, including *PD-1*, were more expressed in the low SHMRGPI group. Based on the TIDE scores to predict the potential clinical efficacy of immunotherapy in different SHMRGPI groups, it was found that the high SHMRGPI group was more likely to exhibit T cell dysfunction and exclusion and might have poor response when receiving immunotherapy (15). Therefore, we speculate that SHMRGPI can be used as a potential tool to screen patients with EEC who are suitable for immunotherapy.

We found differences in mutation frequency between SHMRGPI groups by analyzing somatic mutation data. Calculation of TMB revealed differences close to the statistical threshold in TMB levels between SHMRGPI groups. Subsequent analysis of data based on microsatellite instability status revealed a higher proportion of MSI-H in the low SHMRGPI group and a lower SHMRGPI in patients with MSI-H than in patients with MSS. Data from Keynote 028 and Keynote 158 supported the view that patients with TMB-H, MSI-H, and PD-1/PD-L1 positive relapsed or metastatic endometrial cancer can benefit from immunotherapy (29–31). In our study, the low SHMRGPI group with high TMB levels and a high proportion of MSI-H was more likely to benefit from immunotherapy. This result is consistent with the aforementioned results of SHMRGPI and immune correlation analysis.

Whether the encouraging results of poly (ADP-ribose) polymerase inhibitors (PARPi) in maintenance therapy for ovarian cancer can be replicated in the management of patients with endometrial cancer is a common concern among gynecologic oncologists (10, 32, 33). The results of the drug sensitivity analysis of gynecologic antineoplastic agents showed that PARPi (including olaparib, niraparib and talazoparib) differed in drug sensitivity between the SHMRGPI groups. In breast cancer, estrogen was found to enhance the cytotoxicity of PARP inhibitors on ER-positive tumor cells, resulting in inhibition of cell growth (34). In EEC, the ability of SHMRGPI to screen potential PARPi beneficiaries and the mechanism of correlation between SHMRG and PARPi remain to be further confirmed.

A limitation of this study is that the data used to build the model were obtained from a retrospective database and the findings are potentially susceptible to bias. Inferences based on the results of immune and drug sensitivity analysis need to be supported by additional experimental evidence.

## 5 Conclusions

In this study, we developed a prognostic model and analyzed it with respect to clinical, somatic mutation, immune and drug sensitivity. Focusing on biomarkers shared by endometrial cancer and breast cancer, it provides a new idea for the precise treatment of patients with EEC.

## Data availability statement

The original contributions presented in the study are included in the article/**Supplementary Material**. Further inquiries can be directed to the corresponding author.

## Author contributions

JD and CL participated throughout the conception, data analysis, and manuscript writing of the study. JY was primarily involved in the writing of the study. YW was involved in the conception of the study and review of the manuscript. All authors contributed to the article and approved the submitted version.
